# Real-Time Translocation and Function of PKCβII Isoform in Response to Nociceptive Signaling via the TRPV1 Pain Receptor

**DOI:** 10.3390/ph4111503

**Published:** 2011-11-11

**Authors:** Sravan Mandadi, Patricia J. Armati, Basil D. Roufogalis

**Affiliations:** 1 Hotchkiss Brain Institute, University of Calgary, 3330 Hospital Drive NW, Calgary, AB T2N4N1, Canada; 2 Brain Mind Research Institute and the Nerve Research Foundation, University of Sydney, Sydney, NSW 2006, Australia; 3 Faculty of Pharmacy, University of Sydney, Room 341, Pharmacy and Bank Building A15, Sydney, NSW 2006, Australia

**Keywords:** pain, protein kinase C, transient receptor potential vanilloid-1, real-time translocation, dorsal root ganglion neurons, nociceptive signaling

## Abstract

Serine/threonine protein kinase C βII isoform (PKCβII) or the pain receptor transient receptor potential vanilloid 1 (TRPV1) have been separately implicated in mediating heat hyperalgesia during inflammation or diabetic neuropathy. However, detailed information on the role of PKC βII in nociceptive signaling mediated by TRPV1 is lacking. This study presents evidence for activation and translocation of the PKC βII isoform as a signaling event in nociception mediated by activation of TRPV1 by capsaicin. We show that capsaicin induces translocation of cytosolic PKCβII isoform fused with enhanced green fluorescence protein (PKCβII-EGFP) in dorsal root ganglion (DRG) neurons. We also show capsaicin-induced translocation in Chinese Hamster Ovarian (CHO) cells co-transfected with TRPV1 and PKCβII-EGFP, but not in CHO cells expressing PKCβII-EGFP alone. By contrast, the PKC activator phorbol-12-myristate-13-acetate (PMA) induced translocation of PKCβII-EGFP which was sustained and independent of calcium or TRPV1. In addition PMA-induced sensitization of TRPV1 to capsaicin response in DRG neurons was attenuated by PKCβII blocker CGP 53353. Capsaicin response via TRPV1 in the DRG neurons was confirmed by TRPV1 antagonist AMG 9810. These results suggested a novel and potential signaling link between PKCβII and TRPV1. These cell culture models provide a platform for investigating mechanisms of painful neuropathies mediated by nociceptors expressing the pain sensing gene TRPV1, and its regulation by the PKC isoform PKCβII.

## 1. Introduction

Hypersensitivity to nociception or hyperalgesia in many peripheral neuropathies is currently undertreated or requires complex drug treatment with significant side effects. These conditions represent a huge cost to health budgets worldwide. One of the main problems is our incomplete knowledge of the underlying mechanisms and individual responses to drug regimes. One of the defined and important mechanisms is an interaction between the serine/threonine protein kinase C (PKC) [1,2] and the transient receptor potential vanilloid 1 (TRPV1). TRPV1 is the molecular integrator of noxious heat (>42 °C), the hot chilli pepper ingredient capsaicin and tissue acidosis [3,4], and is characteristic of a nociceptive subset of dorsal root ganglion (DRG) neurons [5]. Nociceptive subsets of DRG neurons in culture have been a particularly useful tool for the investigation of signaling mechanisms associated with hyperalgesia. A classical phenomenon that occurs during PKC activation is its translocation from the cytosol to the plasma membrane, where PKC phosphorylates and modulates ion channel function [6-14]. Such translocation was suggested as a mechanism for PKCε-mediated sensitization in response to noxious heat [15,16]. The PKCε-mediated phosphorylation of TRPV1 [17], is proposed to correlate with TRPV1-mediated heat hyperalgesia in models of inflammatory pain [18,19]. Among other isoforms, PKCδ is known to be expressed in the cytosol of rat DRG neurons [15]. Although PKCδ is translocated from the cytosol to the plasma membrane, its role in sensitizing the TRPV1 nociceptor responsiveness to noxious heat has been ruled out [15]. Of particular interest is that two other isoforms, PKCβI or PKCβII, are more extensively localized on the cell membrane than in the cytosol [15]. Not surprisingly, in the same study, activation of the PKCβI or PKCβII by the potent PKC activator, phorbol-12-myristate-13-acetate (PMA) [20], was reported not to alter this localization [15]. Interestingly and of relevance is that in a model of inflammatory pain where bradykinin mediates activation of PKC, neither of the two PKCβ isoforms mediated sensitization to noxious heat [15]. However, PKCβII did play a role in mediating heat hyperalgesia in an inflammation model induced by complete Freund's adjuvant (CFA) [21]. Also, in painful diabetic neuropathy an increased membrane localization and activity of PKCβII has been implicated in inducing mechanical hyperalgesia [22] as well as heat hyperlagesia [23-25]. There is general consensus that the underlying mechanism for heat hyperalgesia does involve PKC-mediated sensitization of TRPV1 [24,26,27]. However, the specific isoforms of PKC that may be involved in sensitization of TRPV1 during diabetic neuropathy remains unresolved. To the best of our knowledge there have been no reports linking activation and translocation of PKCβII during nociceptive signaling via the pain receptor TRPV1. The current study therefore addresses the relevant issues related the localization and real-time translocation of cytosolic PKCβII in relation to TRPV1 activity. We also show a novel functional role for PKCβII in sensitizing TRPV1 in DRG neurons.

In a previous report we demonstrated the co-expression of PKCβII with TRPV1 in a subset of cultured DRG neurons a significant subset of which were TRPV1 positive and expressed cytosolic PKCβII [28]. Since an increase in intracellular calcium [Ca^2+^]i can activate and translocate PKCβ isoforms to subcellular sites [29], we tested the hypothesis that the cytosolic PKCβII isoform will be activated and translocated to the plasma membrane following an increase in [Ca^2+^]i, known to occur via TRPV1 activation [3]. Here we used a culture model of DRG neurons transiently transfected with PKCβII-EGFP. Capsaicin, the potent and selective agonist of TRPV1 [3,18] was used to activate capsaicin-sensitive DRG neurons. Further evidence for TRPV1 mediated translocation of the PKCβ isoforms was examined in Chinese hamster ovarian (CHO) cells transiently transfected with PKCβII-EGFP or transiently co-transfected with PKCβII-EGFP and TRPV1. Translocation of the PKCβII isoform independent of increase in [Ca^2+^]i or TRPV1 was tested using the phorbol ester, PMA. Moreover, PMA-induced sensitization of capsaicin response in DRG neurons was attenuated by PKCβ blocker CGP 53353. Capsaicin response in the presence of PMA in DRG neurons was completely blocked by the TRPV1 antagonist AMG 9810 thus confirming that the capsaicin response in DRG neurons was mediated via TRPV1. Our study shows that these cell culture models are useful for defining the sub-cellular localization, movement and functional role of PKCβII isoforms in relation to TRPV1 signaling. These models can be further applied to define the role of PKCβII isoform in heat hyperalgesia associated with TRPV1 under pathological conditions including painful neuropathies especially diabetic neuropathy.

## 2. Materials and Methods

### 2.1. Isolation and Culture of Rat DRG Neurons

This study was approved and carried out in accordance with the guidelines of the Animal Ethics Committee, University of Sydney (# L24/11-99/3/3047). DRG neurons were obtained as described earlier [30]. Briefy, isolated DRG from neonatal (three- to five-day-old) Sprague Dawley rats (Laboratory Animal Services of the University of Sydney) were incubated in Hank's CMF saline with 0.05% collagenase and 0.25% trypsin for 25 min at 37 °C. Neurons were obtained by trituration of dissociated DRG cells with fire-polished Pasteur pipettes of decreasing diameter and afterwards the cellular suspension was washed twice in Dulbecco's modified Eagle medium supplemented with 10% fetal calf serum and 2 mM glutamine. Freshly isolated neurons were plated onto collagen-coated coverslips and cultured in Neurobasal medium with B27 supplement, 50 ng/mL 2.5S nerve growth factor, 2 mM glutamine, 100 U/mL penicillin and 100 mg/mL streptomycin. DRG neurons were plated at 70-80% confluence on 19 mm collagen-coated coverslips were kept under above culture conditions at 37 °C with 5% CO_2_. One day old cultures on the coverslips were used for experiments.

### 2.2. Culture of CHO Cells

CHO cells were maintained in cell culture flasks containing Ham's F-12 medium (supplemented with 10% fetal bovine serum, 100 U/mL penicillin, 100 μg/mL streptomycin) under standard culture conditions of 37 °C and 5% CO_2_ in a humidified incubator. The cells were then re-plated at 70-80% confluence on 19 mm glass coverslips in 12-well tissue culture plates under similar culture conditions. One day old cultures on the coverslips were used for experiments.

### 2.3. Transient Transfection of PKCβII-EGFP in DRG Neurons or CHO Cells

One day old cultures of DRG neurons or CHO cells were transiently transfected with pPKCβ-EGFP containing PKCβII subunit (Clontech Laboratories, Inc. Palo Alto, CA, USA), using the Effectene Transfection Reagent (QIAGEN, Valencia, CA, USA) protocol for 12-well culture plates. 48 h post-transfection, the DRG neurons or CHO cells were examined by confocal laser scanning microscopy for translocation of PKCβII-EGFP in real-time.

### 2.4. Transient Co-Transfection of PKCβII-EGFP with TRPV1 in CHO Cells

CHO cells cultured as described above were used for the co-transfection experiments. One day old cultures were transiently co-transfected with 0.5 μg of PKCβII-EGFP plus 0.5 μg of TRPV1 per well, using Lipofectamine 2000 *as per* the manufacturer's 12-well plate protocol (Invitrogen Carlsbad, CA, USA). 48 h post-transfection, the CHO cells were examined by confocal laser scanning microscopy for translocation of PKCβII-EGFP in real-time.

### 2.5. Real-Time Translocation of PKCβII-EGFP

Coverslips with transiently transfected DRG neurons or CHO cells cultured as described above were placed onto a chamber and sealed with wax. The chamber was the placed on a TCS SP2 System (Leica) confocal microscope attached to a rapid sample perfusion system. For each experiment, fluorescence was recorded from individual cell bodies and each image was acquired at 2 seconds intervals with a HC × PL APO 63x/1.20 W CORR objective. The chamber was continuously perfused with a solution consisting of 140 mM NaCl, 2 mM CaCl_2_, 5 mM KCl, 20 mM HEPES, 10 mM glucose, pH 7.4. Experiments where nominal calcium free solutions were used consisted of 140 mM NaCl, 10 μM BAPTA, 200 μM EGTA, 5 mM KCl, 20 mM HEPES, 10 mM glucose, pH 7.4. The chamber had a volume of 1,000 μL and solution changes were complete within 20 s (duration of drug applications). An Argon-Krypton laser was used as light source to excite EGFP. The fluorescence changes were represented as the ratio ΔF/F (ΔF = Change in fluorescence intensity; F = Initial fluorescence intensity) versus time (seconds). Fluorescence signal was obtained by drawing a region of interest on the visible area of the single cell body cytosol. Change in fluorescence thus represented a decrease in PKCβII-EGFP in the cytosol when the isoform translocated to the plasma membrane. The fluorescence would return to baseline following relocation of PKCβII-EGFP from the plasma membrane to the cytosol. All experiments were performed at RT (20-22 °C).

### 2.6. Calcium Signaling

DRG neurons (1 × 10^4^) were plated onto 96-well microplates (Perkin Elmer Waltham, MA, USA) and maintained in culture medium as described in section 2.1. One day old cultures were incubated at 37 °C with 5% CO_2_ for 30 min in Fluo-4/AM no wash calcium indicator (Invitrogen) and assayed for calcium signaling responses on a victor X4 fluorescent plate reader (PerkinElmer Life Sciences). Calcium signals were monitored (one count every 0.5 s for 2 min after drug application) using an excitation wavelength of 480 nm and an emission wavelength recorded at 530 nm. Changes in fluorescence were expressed as a percentage of the ratio ΔF/F (ΔF = Change in fluorescence intensity; F = Initial fluorescence intensity). The calcium transient traces were truncated to represent the first 0.5 min out of the 2 min of calcium transients monitored under each experimental condition. All experiments were performed at RT (20–22 °C).

### 2.7. Chemicals

Capsaicin and phorbol-12-myristate-13-acetate were from Sigma (St. Louis, MO, USA); CGP 53353 and AMG 9810 were from Tocris Bioscience (Ellisville, MI, USA).

## 3. Results

### 3.1. Real-Time Translocation of Transiently Transfected PKCβII-EGFP in DRG Neurons

**Figure 1.**
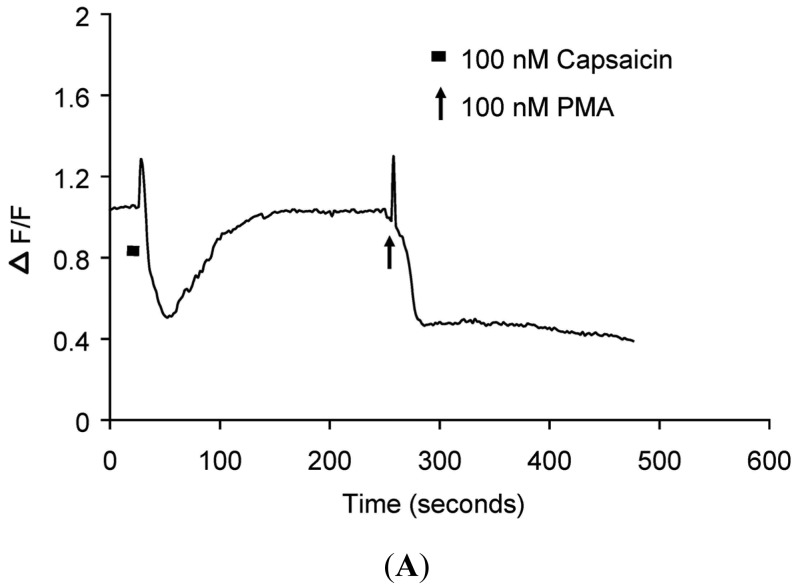

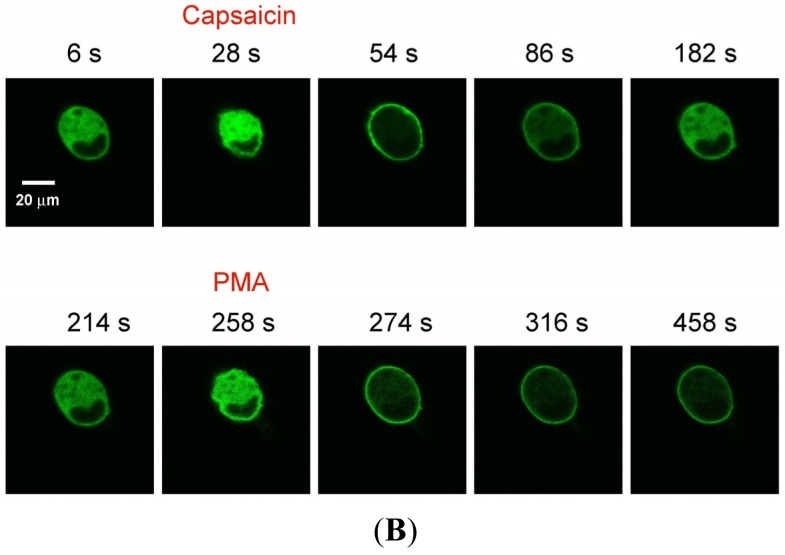
Translocation kinetics of PKCβII-EGFP in a single cell body of capsaicin-sensitive DRG neuron.

**Figure 2.**
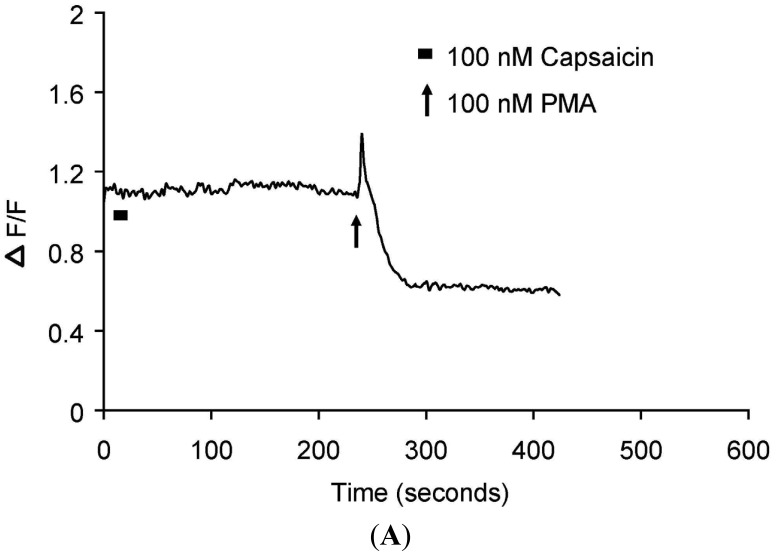

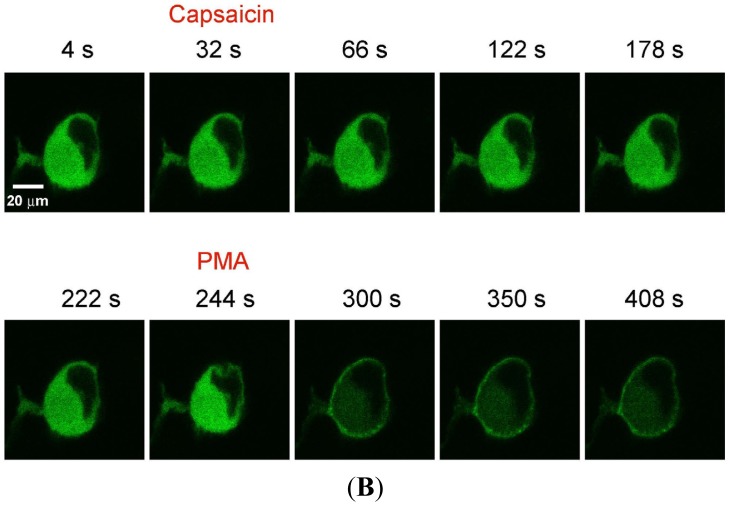
Translocation kinetics of PKCβII-EGFP in a single cell body of capsaicin-insensitive DRG neuron.

Real-time translocation of cytosolically expressed PKCβII-EGFP was examined separately in individual DRG neurons. In the presence of extracellular Ca^2+^, 100 nM capsaicin induced a transient and reversible (<3 min) translocation of PKCβII-EGFP (Figure 1A–B and Supplementary Data video file Capsaicin response in DRG neurons) in a subset of small to medium diameter DRG neurons (5-30 μm). However, a subset of DRG neurons, with cell body of diameter 5-30 μm or greater than 35 μm was not capsaicin responsive (Figure 2A–B). 100 nM PMA applied at the end of each experiment induced a sustained translocation (>3 min) (Figure 1A–B, 2A–B and Supplementary Data video file PMA response in DRG neurons). PMA-induced translocation was independent of cell body diameter and capsaicin sensitivity (Figures 1 and 2). In DRG neurons expressing only plasma membrane-associated PKCβII-EGFP, neither capsaicin nor PMA change its localization (data not shown).

### 3.2. TRPV1 Mediated Real-Time Translocation of Transiently Transfected PKCβII-EGFP in CHO Cells

**Figure 3.**
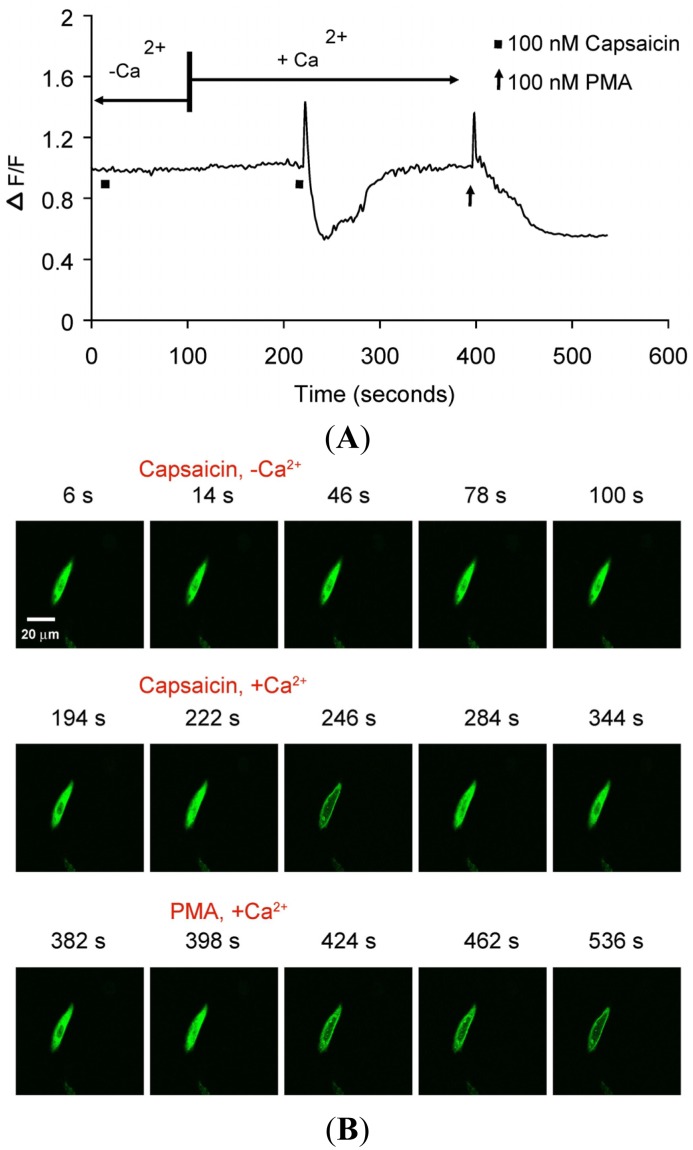
Translocation kinetics of PKCβII-EGFP in a single cell body of CHO cell from a culture co-transfected with PKCβII-EGFP and TRPV1.

TRPV1-mediated translocation of PKCβII was further investigated in real-time using CHO cells transiently transfected with PKCβII-EGFP or transiently co-transfected with PKCβII-EGFP plus TRPV1. 100 nM capsaicin induced transient and reversible (<3 min) calcium-dependent translocation of PKCβII-EGFP (Figures 3A–B and Supplementary Data video file Capsaicin response in CHO cells). 100 nM PMA induced sustained PKCβII-EGFP translocation (>3 min), (Figures 3A–B and Supplementary Data video file PMA response in CHO cells).

**Figure 4.**
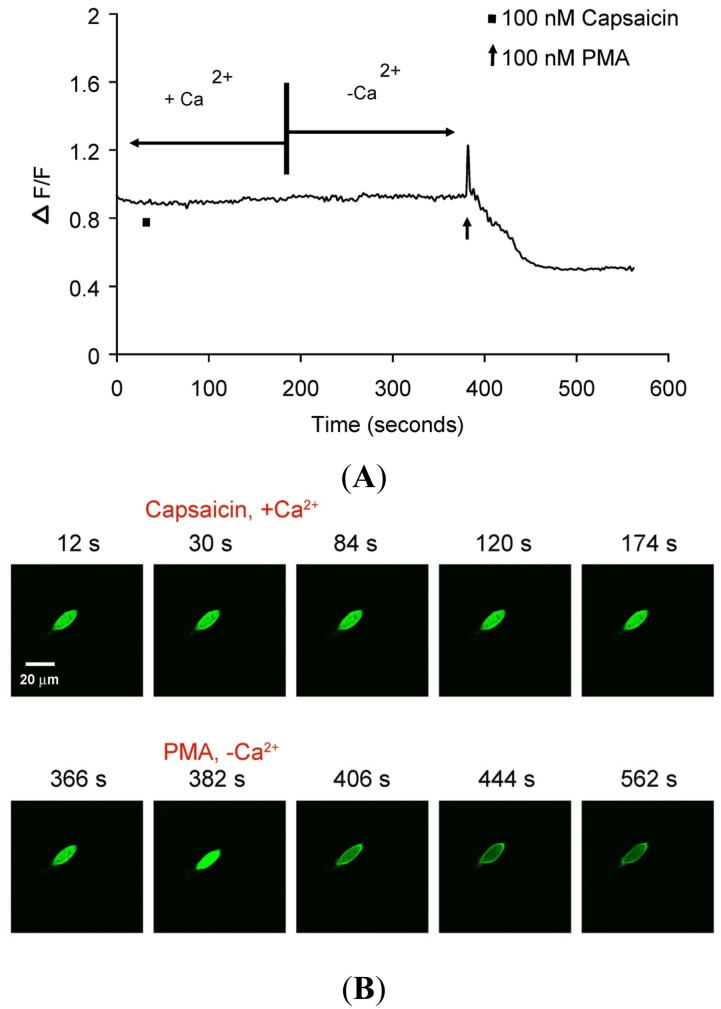
Translocation kinetics of PKCβII-EGFP in a single cell body of CHO cell from a culture transfected with PKCβII-EGFP only.

There was no translocation in response to capsaicin stimulus in cells expressing only PKCβII-EGFP but not TRPV1 (Figures 4A–B) indicating that capsaicin-induced translocation is TRPV1-dependent. 100 nM PMA induced sustained PKCβII-EGFP translocation (>3 min), independent of TRPV1 or calcium (Figures 4A–B and Supplementary Data video file PMA response in CHO cells).

### 3.3. PKCβII Mediated Sensitization of Capsaicin Response in DRG Neurons

**Figure 5.**
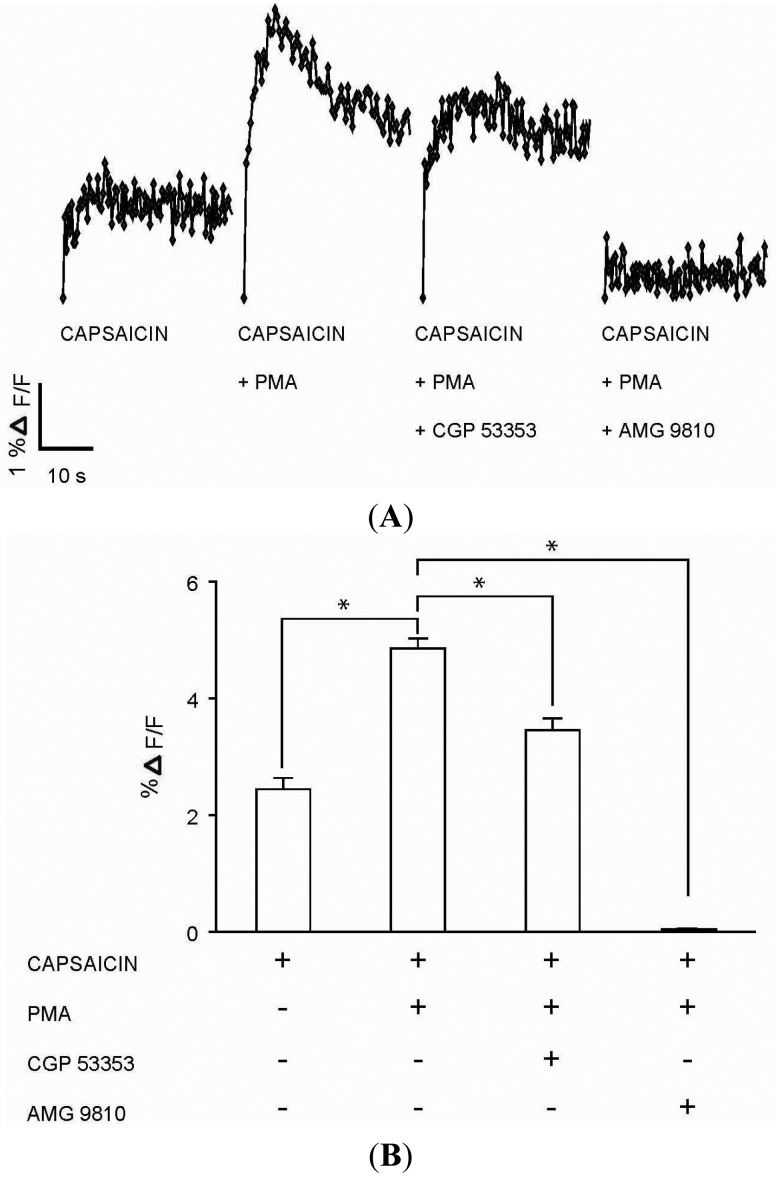
PKCβII sensitizes TRPV1-mediated calcium signaling in DRG neurons.

The functional role for PKCβII in modulating calcium signaling via TRPV1 was tested in DRG neurons. Capsaicin (100 nM) response (Figures 5A–B) in DRG neurons was significantly enhanced in the presence of PMA (100 nM) (Figures 5A–B). The enhanced response to capsaicin induced by PMA was significantly attenuated in the presence of PKCβII blocker CGP 53353 (100 nM) (Figures 5A–B). In addition, the response to capsaicin in the presence of PMA was completely inhibited by TRPV1 antagonist AMG 9810 (1 μM) (Figures 5A–B), suggesting capsaicin action on TRPV1. Control experiments which included DRG neurons exposed to only PMA (100 nM), CGP 53353 (100 nM) or AMG 9810 (1 μM) did not evoke a calcium response (data not shown). These results suggested a potential role for PKCβII in the modulation of the TRPV1 signaling in DRG neurons.

## 4. Discussion

Diabetic neuropathy is characterised by altered thermal sensitivity of primary nociceptive afferents [23-25], and consists of two phases [31]. A first transient phase of thermal hyperalgesia is followed by a prolonged second phase of thermal hypoalgesia [31]. The increased expression and sensitivity of TRPV1 in DRG neurons may be the mechanism for the early transient phase of thermal hyperalgesia [24,26,27]; the mechanism behind thermal hypoalgesia is suggested to be due to a severe down-regulation of TRPV1 expression [24]. The mechanism for thermal hyperalgesia to noxious heat is suggested to involve PKC-mediated sensitization of TRPV1 [24,26,27]. However, the role of specific PKC isoform/s mediating the TRPV1 sensitization during the first phase of diabetic neuropathy is not well understood. Increased activity of PKCβII isoforms as an important event in thermal hyperalgesia was reported by Anand *et al.* [23]. However, current understanding following the cloning of TRPV1 [3] makes it unlikely that the activity of PKCβII alone is involved in hyperalgesia. Hence, despite several studies, knowledge of specific mechanisms in altered thermal sensitivity during diabetic neuropathy remains incomplete. As the methodological tools used in earlier studies could not delineate which molecules interacted during altered thermal sensitivity, we addressed this issue by characterizing changes in the localization and translocation of PKCβII isoforms with respect to TRPV1 activation - the regulators known to be important in altered thermal sensitivity [24,26,27].

Using cultured DRG neurons, PKCβII was shown to be localized mainly at the plasma membrane rather than the cytosol as reported by Cesare *et al.* [15]. Perhaps not surprisingly, the same study also showed that PKCβII isoforms did not translocate after activation by PMA [15] which activates and translocates other PKCβ isoforms [20]. However, the co-expression of PKC isoforms with TRPV1 was not determined, even though noxious heat was used as a stimulus to represent TRPV1 activation or sensitization [15]. Consistent with Cesare *et al.* [15] we have previously demonstrated plasma membrane localization of PKCβI or PKCβII in a specific subpopulation of the neonatal rat DRG neurons [28]. In addition, we determined that subpopulations of DRG neurons also have PKCβI and PKCβII localized in the cytosol [28] and for the first time defined that the subpopulations of DRG neurons expressing PKCβ isoforms also co-express TRPV1 [28].

We now provide evidence that addresses the issue of possible false negative results obtained by Cesare *et al.* where translocation of PKCβ isoforms could not be shown using immunocytochemistry [15]. In our study we did not use immunocytochemistry to investigate translocation of the endogenously expressed PKCβII isoform for two reasons: (i) localization of the PKCβ isoforms at the plasma membrane or cytosol of naive DRG neurons may create the caveat of false negative or false positive results for a translocation event; (ii) immunocytochemistry cannot capture in real-time the PKC translocation kinetics induced by different kinds of stimuli applied to individual neurons. This is particularly important as PKC translocation can be sustained, oscillatory, or transient, depending on the isozyme, cell type, and stimulus [13,14,29,32-37]. We therefore defined the real-time translocation of PKCβII-EGFP from the cytosol to the plasma membrane in a subpopulation of DRG neurons with cytosolic localization of the PKC. Capsaicin, the selective agonist of TRPV1 [3,18], induced a transient translocation while PMA induced sustained translocation of the PKC-EGFP's from cytosol to plasma membrane. The capsaicin-induced transient translocation of PKCβ-EGFPs correlates with the transient rise in intracellular calcium [Ca^2+^]i known to be induced by capsaicin via TRPV1 [3]. While the PKCβ isoforms are activated and translocated from the cytosol to the plasma membrane by increases in [Ca^2+^]i, the translocation is reversed when the increase in [Ca^2+^]i is reversed [29,32]. In contrast, the PMA-induced sustained translocation of the PKCβII-EGFPs is consistent with the known sensitivity and kinetics of translocation by PKCβ isoforms in response to the phorbol esters [29,32]. It was expected that the subset of DRG neurons insensitive to capsaicin-induced translocation of cytosolic PKCβII-EGFP, might not express TRPV1. Also, the PMA induced translocation of cytosolic PKCβII-EGFP was expected to be independent of the capsaicin-sensitivity and or TRPV1 expression in DRG neurons.

We confirmed the signaling event of capsaicin- or PMA-mediated translocation of cytosolic PKCβII-EGFP depended on the expression of TRPV1 in CHO cells. The TRPV1-mediated signaling was confirmed by comparing capsaicin-induced translocation response in CHO cells transiently transfected with PKCβII-EGFP alone or CHO cells co-transfected with the combination of PKCβII-EGFP and TRPV1. A transient translocation of PKCβII-EGFP from the cytosol to the plasma membrane in response to capsaicin was observed in CHO cells co-transfected with TRPV1 and PKCβII-EGFP, but not in those transfected with PKCβII-EGFP alone. The transient nature of capsaicin-induced translocation was similar to that observed in the DRG neurons. Also, there was no capsaicin-induced translocation in CHO cells expressing both TRPV1 and PKCβII-EGFP in the absence of extracellular Ca^2+^. These results supported the concept that the transient PKCβII-EGFP translocation was due to a transient increase in [Ca^2+^]i influx induced by the capsaicin activation of TRPV1 [3]. In contrast, PMA-induced a prolonged cell membrane association of the translocated PKCβII-EGFP that was independent of TRPV1 expression and extracellular Ca^2+^.

Finally, functional consequence of PKCβII activation and translocation upon TRPV1 signaling was confirmed in DRG neurons that respond to capsaicin. A sensitization of the capsaicin response in DRG neurons induced by the phorbol ester PMA was attenuated by CGP 53353, a specific blocker of PKCβII [39-41]. Also a specific TRPV1 antagonist completely blocked capsaicin response in DRG neurons suggesting its action via TRPV1. The PMA-induced sensitization was however not completely blocked by the PKCβII inhibitor CGP 53353. The residual sensitization by PMA seen in DRG neurons in the presence of PKCβII inhibitor suggests a role for other PKC isoforms [28]. It would be interesting to see if the potential role of PKCβII in sensitizing TRPV1 signaling is specific to painful diabetic neuropathy. Future studies in diabetic models of thermal hyperalgesia are warranted.

## 5. Conclusions

In conclusion, we have shown the activation, translocation and novel function of PKCβII isoform seen in the capsaicin-sensitive nociceptive DRG neurons. This signalling event may play an essential role in the activation of the pain receptor TRPV1 as seen in CHO cells. While PKCβII or TRPV1 have separately been implicated in altering thermal sensitivity of nociceptive primary afferents during diabetic neuropathy, our results support a role for both in concert with each other. Future investigations using such cell culture assay models as presented here should provide the basis for possible therapeutic leads for treating pain associated with inflammatory conditions and in particular painful diabetic neuropathies now of epidemic proportions worldwide [38]. For example, while DRG neurons would serve as a proof of principle bio-assay in native cells, the heterologous expression system using CHO cells may provide a more effective and high-throughput bio-assay to investigate mechanisms or to screen and develop molecules targeting PKC and/or TRPV1 signaling.

## Figures and Tables

(**A**) Representative trace showing effect of capsaicin and PMA on translocation of cytosolically expressed PKCβII-EGFP to the cell membrane in a single cell body of DRG neuron in the presence of 2 mM extracellular Ca^2+^. 100 nM capsaicin induced reversible translocation of PKCβII-EGFP; 100 nM PMA induced a sustained translocation of PKCβII-EGFP which was irreversible for longer than 3 minutes. Changes in fluorescence were measured before, during and after drug applications. (n = 4); (**B**) Representative images of translocation of cytosolically expressed PKCβII-EGFP to the cell membrane in a single cell body of DRG neuron. These images correspond to the trace in Figure 1A and the videos in supplementary material. Control condition image (6 s); capsaicin application image (28 s); capsaicin-induced translocation to the cell membrane image (54 s); relocation to cytosol following capsaicin washout images (86 s and 182 s); pre-PMA application image (214 s); PMA application image (258 s); PMA-induced translocation to the cell membrane images (274 s, 316 s and 458 s). All drug applications were made in the presence of 2 mM extracellular Ca^2+^.

(**A**) Representative trace showing effect of capsaicin and PMA on translocation of cytosolically expressed PKCβII-EGFP to the cell membrane in a single cell body of capsaicin-insensitive DRG neuron in the presence of 2 mM extracellular Ca^2+^. 100 nM capsaicin did not induce translocation of PKCβII-EGFP; 100 nM PMA induced a sustained translocation of PKCβII-EGFP which was irreversible for longer than 3 minutes. Changes in fluorescence were measured before, during and after drug applications. (n = 5); (**B**) Representative images of translocation of cytosolically expressed PKCβII-EGFP to the cell membrane in a single cell body of capsaicin-insensitive DRG neuron. These images correspond to the trace in Figure 2A. Control condition image (4 s); capsaicin application image (32 s); no translocation to the cell membrane image by capsaicin (66 s, 122 s and 178 s); pre-PMA application image (222 s); PMA application image (244 s); PMA-induced translocation to the cell membrane images (300 s, 350 s and 408 s). All drug applications were made in the presence of 2 mM extracellular Ca^2+^.

(**A**) Representative trace showing effect of capsaicin and PMA on translocation of cytosolically expressed PKCβII-EGFP to the cell membrane in a single cell body of CHO cells. The CHO-cell was co-transfected with TRPV1 and PKCβII-EGFP. Application of 100 nM capsaicin induced reversible translocation of PKCβII-EGFP co-expressed with TRPV1 in the presence of 2 mM extracellular Ca^2+^ but not in its absence. Application of 100 nM PMA at the end of experiment induced a sustained translocation of both PKCβII-EGFP, which was sustained for longer than 3 minutes and independent of TRPV1 and extracellular Ca^2+^. (n = 10); (**B**) Representative images of translocation of cytosolically expressed PKCβII-EGFP to the cell membrane in a single CHO cell body. These images correspond to the trace in Figure 2A and the videos in supplementary material. Control condition (minus extracellular Ca^2+^) image (6 s); capsaicin application (minus extracellular Ca^2+^) image (14 s); no translocation by capsaicin (minus extracellular Ca^2+^) images (46 s, 78 s and 100 s); control condition (plus 2 mM extracellular Ca^2+^) image (194 s); capsaicin application (plus 2 mM extracellular Ca^2+^) image (222 s); capsaicin-induced translocation to cell membrane (plus 2 mM extracellular Ca^2+^) image (246 s); relocation to cytosol following capsaicin washout images (284 s and 344 s); pre-PMA application image (382 s); PMA application image (398 s); PMA-induced translocation to the cell membrane images (424 s, 462 s and 536 s). The PMA application was made in the presence of 2 mM extracellular Ca^2+^.

(**A**) Representative trace showing effect of capsaicin and PMA on translocation of cytosolically expressed PKCβII-EGFP to the cell membrane in a single cell body of CHO cells. The CHO-cell was transfected with PKCβII-EGFP only. Application of 100 nM capsaicin did not induce any translocation of PKCβII-EGFP in the presence of 2 mM extracellular Ca^2+^. Application of 100 nM PMA at the end of experiment induced a sustained translocation of PKCβII-EGFP, which was sustained for longer than 3 minutes and independent of TRPV1 and extracellular Ca^2+^. (n = 10). (**B**) Representative images of translocation of cytosolically expressed PKCβII-EGFP to the cell membrane in a single CHO cell body. These images correspond to the trace in Figure 3A. Control condition (plus 2 mM extracellular Ca^2+^) image (12 s); capsaicin application (plus 2 mM extracellular Ca^2+^) image (30 s); no translocation by capsaicin (plus 2 mM extracellular Ca^2+^) images (84 s, 120 s and 174 s); pre-PMA application (minus extracellular Ca^2+^) image (366 s); PMA application (minus extracellular Ca^2+^) image (382 s); PMA-induced translocation to the cell membrane (minus extracellular Ca^2+^) images (406 s, 444 s and 562 s).

(**A**) Representative traces of calcium transients (% ΔF/F) in DRG neurons exposed to four experimental conditions: capsaicin (100 nM) application; capsaicin (100 nM) application following incubation with PMA (100 nM) for 1 minute; capsaicin (100 nM) application after incubations first with PKCβII blocker CGP 53353 (100 nM) for 3 minutes followed by PMA (100 nM) for 1 minute; and capsaicin (100 nM) application after incubations first with TRPV1 antagonist AMG 9810 (1 μM) for 1 minute followed by PMA (100 nM) for 1 minute. (**B**) Summary bar graph of calcium transients (% ΔF/F) as observed in DRG neuronal cultures exposed to the four experimental conditions as described in Figure 4A. The % ΔF/F ± SEM values for the four experimental conditions: Capsaicin (2.44 ± 0.19%); capsaicin + PMA (4.86 ± 0.17%); capsaicin + PMA + CGP 53353 (3.46 ± 0.20%); capsaicin + PMA + AMG 9810 (0.03 ± 0.02%). (* indicates p < 0.001, ANOVA; n = 4).

## Supplementary Materials

Supplementary materials can be accessed at: http://www.mdpi.com/1424-8247/4/11/1503/s1.

## Supplementary Files

Supplementary File 1: ZIP-Document (ZIP, 3050 KB)
