# Supplementary material for: Real-Time Translocation and Function of PKCβII Isoform in Response to Nociceptive Signaling via the TRPV1 Pain Receptor
Source: Pharmaceuticals (Basel). 2011 Nov 11;4(11):1503–17. doi: 10.3390/ph4111503 (PMC4060137; doi:10.3390/ph4111503)
Supplement: Supplementary File 1: — ZIP-Document (ZIP, 3050 KB) [file pharmaceuticals-04-01503-s001.zip › Mandadi etal Supplementary data/pharmaceuticals-10602-supplementary-videos legends.docx]

**Video Legends**

**Videos showing real-time translocation of PKCβII-EGFP in the same single cell body of DRG neuron**

Real-time translocation of PKCβII-EGFP in response to 100 nM capsaicin **(Capsaicin response in DRG neuron)** and 100 nM PMA **(PMA response in DRG neuron)** in the presence of 2 mM extracellular Ca^2+^. 100 nM capsaicin induced reversible translocation; 100 nM PMA induced a sustained translocation. [Note: The video files have been edited from their original form to show
real-time translocation kinetics representative of response to individual stimuli].

**Videos showing real-time translocation of PKCβII-EGFP in the same single cell body of CHO cell transiently co-transfected with PKCβII-EGFP and TRPV1**

Real-time translocation of PKCβII-EGFP in response to 100 nM capsaicin **(Capsaicin response in CHO cell)** and 100 nM PMA **(PMA response in CHO cell)** in the presence of 2 mM extracellular Ca^2+^. 100 nM capsaicin induced reversible translocation; 100 nM PMA induced a sustained translocation. [Note: The video files have been edited from their original form to show real-time translocation kinetics representative of response to individual stimuli].
